# The agreement between fasting glucose and markers of chronic glycaemic exposure in individuals with and without chronic kidney disease: a cross-sectional study

**DOI:** 10.1186/s12882-020-1697-z

**Published:** 2020-01-30

**Authors:** Cindy George, Tandi E. Matsha, Marizna Korf, Annalise E. Zemlin, Rajiv T. Erasmus, Andre P. Kengne

**Affiliations:** 1 Non-Communicable Diseases Research Unit, South African Medical Research Council, Francie van Zijl Drive, Parow Valley, Cape Town, South Africa; 2 SAMRC/CPUT/Cardiometabolic Health Research Unit, Department of Biomedical sciences, Faculty of Health and Wellness Sciences, Cape Peninsula University of Technology, Bellville, South Africa; 3 Division of Chemical Pathology, Faculty of Medicine and Health Sciences, National Health Laboratory Service (NHLS) and University of Stellenbosch, Cape Town, South Africa; 4 Department of Medicine, University of Cape Town, Cape Town, South Africa

**Keywords:** Chronic kidney disease, Fructosamine, Glucose tolerance, Glycated albumin, Haemoglobin A1c

## Abstract

**Background:**

To assess whether the agreement between fasting glucose and glycated proteins is affected by chronic kidney disease (CKD) in a community-based sample of 1621 mixed-ancestry South Africans.

**Methods:**

CKD was defined as an estimated glomerular filtration rate < 60 ml/min/1.73 m^2^. Fasting plasma glucose and haemoglobin A1c (HbA1c) concentrations were measured by enzymatic hexokinase method and high-performance liquid chromatography, respectively, with fructosamine and glycated albumin measured by immunoturbidimetry and enzymatic method, respectively.

**Results:**

Of those with CKD (*n* = 96), 79, 16 and 5% where in stages 3, 4 and 5, respectively. Those with CKD had higher levels of HbA1c (6.2 vs. 5.7%; *p* < 0.0001), glycated albumin (15.0 vs. 13.0%; *p* < 0.0001) and fructosamine levels (269.7 vs. 236.4 μmol/l; *p* < 0.0001), compared to those without CKD. Higher fasting glucose levels were associated with higher HbA1c, glycated albumin and fructosamine, independent of age, gender, and CKD. However, the association with HbA1c and glycated albumin differed by CKD status, at the upper concentrations of the respective markers (interaction term for both: *p* ≤ 0.095).

**Conclusion:**

Our results suggest that although HbA1c and glycated albumin perform acceptably under conditions of normoglycaemia, these markers correlate less well with blood glucose levels in people with CKD who are not on dialysis.

## Background

Chronic kidney disease (CKD) is estimated to affect about 10% of the general adult population and is even more prevalent in diabetic patients [1, 2]. Indeed, 20–40% of individuals with diabetes have moderate to severe CKD, ranking diabetes as the leading cause of end-stage renal disease (ESRD) and an important risk factor for morbidity and mortality in dialysis patients [3].

It is known that good glycaemic control predicts better clinical outcomes for patients with diabetes, by limiting morbidity and mortality associated with cardiovascular complications and end-organ damage [4, 5]. Traditionally, sequential measurements of blood glucose and/or haemoglobin A1c (HbA1c) (reflecting glycaemic control of the preceding 2–3 months) have been used to monitor glycaemia in patients with diabetes [6]. However, appropriate measures to accurately monitor glucose control in CKD patients remain to be established. Anaemia, which is very common in patients with CKD [7], affects haemoglobin metabolism and thus the level of HbA1c [8]. The predominant cause of anaemia in CKD relates to failure of the kidneys to produce enough erythropoietin, accompanying the fall in glomerular filtration rate (GFR) [7]. Consequently, decreased erythropoiesis leads to increased circulating aged red blood cells (RBCs) and a progressive rise in HbA1c, unrelated to glycaemic control [7, 9]. Contrary, treatment with an erythropoiesis-stimulating agent or iron, results in increased circulating immature RBCs that have a shorter glycaemic exposure time for glycation to occur, resulting in reduced HbA1c levels, with no significant change in mean glucose levels [10]. There are also several other diseases, prevalent in Africa, that affect the clinical utility of HbA1c and for which alternative markers may be necessary, including sickle-cell disease in the more endemic malaria prone regions, as well as HIV/AIDS and tuberculosis [11, 12].

It has been suggested that the relationship between HbA1c and blood glucose concentration is altered as the GFR declines [9]. As such, several alternative indices of glycaemia have been reported, including fructosamine and glycated albumin (GA); both shown to accurately reflect glycaemic control in patients with CKD as they are not impacted by reduced kidney function [13–15]. Fructosamine and GA have shorter half-lives than HbA1c, thus reflecting very recent (1–3 weeks) glycaemic control [16], potentially lessening the confounding effect of shortened RBC survival or high RBC turnover. However, the effect of CKD on the agreement between these indices of glycaemic control has yet to be assessed in the African context; where there is a high frequency of factors affecting HbA1c [11, 12].

The aim of the present study was to determine whether the agreement between fasting blood glucose (FPG) levels and markers of chronic glycaemia exposure (HbA1c, GA and fructosamine) are affected by reduced kidney function in a community-based sample of mixed-ancestry South Africans.

## Methods

### Study population and setting

Data from the Cape Town Vascular and Metabolic Health (VMH) study [17], collected between February 2015 and November 2016, was used in the current cross-sectional analysis. The initial sample included 1647 participants, however 26 participants were excluded due to missing data required to estimate kidney function, including serum creatinine, age or gender. As previously described [17], the participants in the study were all South Africans of mixed-ancestry. The VMH study was approved by the Research Ethics Committees of the Cape Peninsula University of Technology (CPUT) and Stellenbosch University (NHREC: REC—230,408–014 and N14/01/003, respectively) and conducted fully in accordance with the Declaration of Helsinki. As such, procedures were fully explained in the native language of the participant, and voluntarily signed written informed consent was obtained.

### Anthropometric measures and biochemical analysis

As described elsewhere, all interviews and measurements were conducted on the campus of CPUT [18]. Anthropometric measurements were obtained by standard procedures performed three times and the average used for the analysis. Body weight was measured with a calibrated Omron body fat meter HBF-511 digital bathroom scale, height with a stadiometer, and waist circumference (WC) was measured at the level of the narrowest part of the torso, using a non-elastic tape measure. Body mass index (BMI) was calculated by the standard BMI eq.

A standard oral glucose tolerance test (OGTT) was performed by drawing a blood sample after an overnight fast, as well as 2 h after a 75 g oral glucose load, to determine plasma glucose and serum insulin concentrations [19]. All blood samples were analysed by an ISO 15189 accredited Pathology practice (PathCare, Reference Laboratory, Cape Town, South Africa). As previously described [18], plasma glucose levels were measured by enzymatic hexokinase method (Beckman AU, Beckman Coulter, South Africa) and serum insulin with a paramagnetic particle chemiluminescence assay (Beckman DXI, Beckman Coulter, South Africa). HbA1c was analysed with high-performance liquid chromatography (Biorad Variant Turbo, BioRad, South Africa), whereas haemoglobin was measured on a Coulter LH 750 haematology analyzer (Beckman Coulter, South Africa) and fructosamine was determined by immunoturbidimetry on an ABX Pentra 400 autoanalyser (Horiba Medical, USA). Total protein and albumin levels were measured using the Biuret and colourmetric (using bromocresol purple) method, respectively (Beckman AU, Beckman Coulter, South Africa). GA (%) was determined with the quantLab® Glycated Albumin enzymatic assay (Werfen™, Italy). Serum creatinine was measured by the modified Jaffe-Kinetic method (Beckman AU, Beckman Coulter, South Africa). Kidney function was calculated using the serum creatinine-based estimator of glomerular filtration rate (eGFR), namely the 4-variable Modification of Diet in Renal Disease (MDRD) equation [20], with the ethnicity correction factor omitted. The reason for the omission is based on the South African Renal Society CKD guidelines promoting the inclusion of the correction factor only in the case of black Africans.

### Classification of kidney function and co-morbidities

The National Kidney Foundation Disease Outcomes Quality Initiative (NKF-KDOQI) classification [21] was used to classify CKD; with CKD (stage 3–5) defined as an eGFR< 60 ml/min/1.73 m^2^. Glucose levels were used to group participants into glucose tolerance categories according to the WHO criteria [22] as: (1) normal glucose tolerance [FPG < 6.1 mmol/l and 2-h glucose < 7.8 mmol/l]; (2) pre-diabetes including impaired FPG (IFG, 6.1 ≤ FPG < 7.0 mmol/l), impaired glucose tolerance (IGT, 7.8 < 2-h glucose< 11.1 mmol/l) and the combination of both; and (3) type 2 diabetes (T2D) (FPG ≥ 7.0 mmol/l and/or 2-h glucose≥11.1 mmol/l). In addition to the screen-detected T2D, those with a history of previously diagnosed T2D were also grouped as T2D. A BMI greater or equal to 25 kg/m^2^ was classified as overweight and a BMI greater or equal to 30 kg/m^2^ as obese. Anaemia was defined based on the K/DOQI guidelines as haemoglobin level < 13.5 g/dL for men and < 12 g/dL for women [23].

### Statistical analysis

Participant characteristics were summarised as median (25th–75th percentiles) or count and percentages. Group comparisons were analysed by chi-square tests (categorical variables) and Wilcoxon rank-sum tests (continuous variables). Correlations between FPG, HbA1c, GA, and fructosamine were evaluated using Spearman’s rank correlation coefficients (rho, *r*). To test the significant difference between the Spearman correlation coefficients, principles of the Steiger test were used. Robust multiple linear regression models were used to assess the independent association between FPG and the glycaemic indices, while adjusting for age, gender, CKD status and the interaction between CKD status and the glycaemic marker. Further adjustments were made, which included the addition of BMI to the regression models for all the glycaemic markers (Appendix Table 3, Model 1), and haemoglobin (in the model for HbA1c) or serum albumin (in the model for GA) (Appendix Table 3, Model 2). To investigate the interaction between FPG and the glycaemic markers dichotomised by CKD status, predictive margins were estimated, and graphs plotted for each glycaemic marker. The average marginal effect was also computed from the predictive margins (annotated as dy/dx). Similar analysis, as described above, were conducted in a sub-group of participants with confirmed diabetes (*n* = 277) (Appendix Tables 4, 5 and 6 and Appendix Figs. 3 and 4). Statistical analyses were performed using STATA version 15 (Statcorp, College Station, TX) and statistical significance was based on a *p*-value < 0.05, except for interaction tests; which was set at 0.10. This modification of the alpha level to 10% was to assess the effect modification, thus evaluating the magnitude of the association between fasting glucose and the markers of glycaemia by CKD status.

## Results

The general participant characteristics, which have been presented in some detail previously [18], are summarised in Table 1. Briefly, in the sample of 1621 participants, 25.1% were males, with a group median age of 51 years, and 6% of the total sample had CKD (eGFR< 60 ml/min/1.73m^2^). In the group with CKD, 79.2, 15.6 and 5.2% presented with stages 3, 4 and 5 CKD, respectively. Furthermore, CKD was associated with older age (68 vs. 49 years; *p* < 0.0001), a larger WC (99.0 vs. 90.8 cm; *p* < 0.0001) and higher BMI (30.4 vs. 28.2 kg/m^2^; *p* = 0.0035), compared to the participants without CKD. Only 19.8% of those with CKD were of normal weight, compared to 35.3% in those with normal kidney function. Higher fasting and 2-h blood glucose (5.3 vs. 5.0 mmol/l; *p* < 0.0001 and 7.4 vs. 6.0 mmol/l; *p* < 0.0001, respectively) and fasting and 2-h insulin levels (7.6 vs. 6.7 IU/l; *p* = 0.0328 and 58.8 vs. 37.3 IU/l; *p* = 0.0003, respectively) were found in the CKD group compared to those with normal kidney function. Consequently, 19.8 and 38.5% of the CKD participants had IFG/IGT and T2D, respectively. In addition, CKD was coupled with a lower haemoglobin level (12.5 vs. 13.5 g/dL; *p* < 0.0001), compared to those with normal kidney function, with 44.8% of the CKD participants presenting with anaemia. The prevalence of anaemia increased with increasing CKD-stage, from 40.0% at stage 3, to 77.8% at stages 4–5. Participants with CKD had higher levels of HbA1c (6.2 vs. 5.7%; *p* < 0.0001); increasing incrementally for each glycaemic group, namely normoglycaemia [median (25th–75th percentile): 6.0 (5.7–6.2)], IFG/IGT [median (25th–75th percentile): 6.2 (5.9–7.1)] and T2D [median (25th–75th percentile): 7.3 (6.3–8.9)]. Similarly, GA was also higher in those with CKD compared to those without CKD (15.0 vs. 13.0%; *p* < 0.0001), with an incremental increase from normoglycaemia [median (25th–75th percentile): 14.1 (13.4–15.1)], to IFG/IGT [median (25th–75th percentile): 15.3 (14.2–16.3)] and T2D [median (25th–75th percentile): 17.7 (14.9–23.0)]. Finally, the same increased levels of fructosamine was observed in those with CKD with normoglycaemia [median (25th–75th percentile): 245.9 (221.7–363.6)], IFG/IGT [median (25th–75th percentile): 282.3 (248.1–309.5)] and T2D [median (25th–75th percentile): 285.5 (269.7–356.9)], with fructosamine levels higher in those with CKD compared to those with normal kidney function (269.7 vs. 236.4 μmol/l; *p* < 0.0001). Serum albumin levels were similar in those with CKD compared to those without CKD (4.25 vs 4.20 g/dL; *p* = 0.0601).

**Table 1:** Clinical characteristics of the study population overall and by CKD status

Variables	Total (*n* = 1621)	Without CKD (*n* = 1525)	CKD (*n* = 96)	*p*-value
Age (years)	51 (37–61)	49 (36–59)	68 (62–73.5)	< 0.0001
Gender (n,% male)	406 (25.1)	378 (25.4)	19 (19.8)	0.221
Anthropometry				
WC (cm)	91.5 (78.1–103.2)	90.8 (77.5–102.8)	99.0 (89.0–105.8)	< 0.0001
BMI (kg/m^2^)	28.3 (22.8–34.1)	28.2 (22.5–34.1)	30.4 (26.0–36.1)	0.0035
Biochemical analysis and calculations				
Fasting blood glucose (mmol/l)	5.0 (4.6–5.7)	5.0 (4.6–5.6)	5.3 (5.0–7.1)	< 0.0001
2-h glucose (mmol/l)	6.0 (4.9–7.6)	6.0 (4.8–7.5)	7.4 (6.1–9.2)	< 0.0001
Fasting insulin (IU/l)	6.7 (4.2–11.0)	6.7 (4.2–10.9)	7.6 (5.1–12.1)	0.0328
2-h insulin (IU/l)	37.9 (20.5–70.9)	37.3 (19.8–69.7)	58.8 (29.5–105.2)	0.0003
Haemoglobin (g/dL)	13.5 (12.6–14.4)	13.5 (12.6–14.5)	12.5 (11.15–13.45)	< 0.0001
HbA1c (%) (*n* = 1610)	5.8 (5.4–6.3)	5.7 (5.4–6.2)	6.2 (5.9–7.1)	< 0.0001
Albumin (g/dL)	4.24 (4.04–4.41)	4.25 (4.05–4.42)	4.20 (3.99–4.31)	0.0601
GA (%) (*n* = 1504)	13.1 (12.1–14.4)	13.0 (12.1–14.2)	15.0 (13.7–17.7)	< 0.0001
Fructosamine (μmol/l) (*n* = 636)	238.8 (221.1–263.7)	236.4 (220.1–259.1)	269.7 (234.1–304.0)	< 0.0001
Creatinine (μmol/l)	59.0 (52.0–70.0)	59.0 (51.0–68.0)	105.5 (89.0–137.5)	< 0.0001
eGFR (ml/min/1.73m^2^)	–	104.4 (88.4–121.5)	48.5 (34.1–56.2)	< 0.0001
Co-morbidities				
Anaemia (n, %)	311 (19.2)	268 (17.6)	43 (44.8)	< 0.0001
BMI categories (n, %)				0.008
Normal weight	557 (34.4)	538 (35.3)	19 (19.8)	
Overweight	372 (23.0)	345 (22.6)	27 (28.1)	
Obese	692 (42.7)	642 (42.1)	50 (52.1)	
Glucose tolerance categories (n, %)				< 0.0001
Normal glucose tolerance	1048 (64.7)	1009 (66.2)	39 (40.6)	
IFG/IGT	281 (17.3)	262 (17.2)	19 (19.8)	
T2D	277 (17.1)	240 (15.7)	37 (38.5)	

Data is presented as median (25th–75th percentiles) and percentages

*WC* waist circumference, *BMI* body mass index, *HbA1c* glycated haemoglobin, *GA* glycated albumin, *eGFR* estimated glomerular filtration rate, *IFG/IGT* impaired fasting glucose and impaired glucose tolerance, *T2D* type 2 diabetes mellitus

**Fig. 1:**
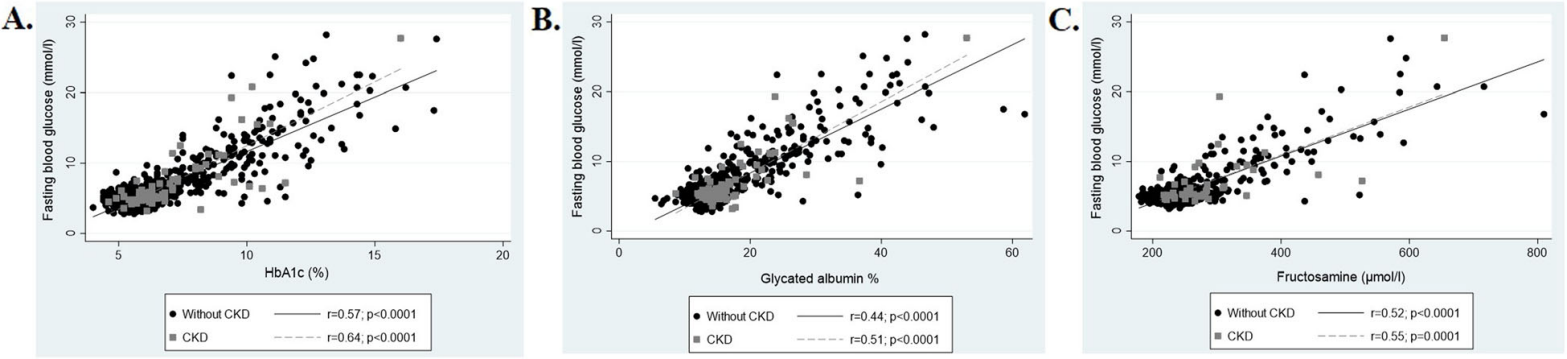
Correlation between fasting glucose, **a** HbA1c, **b** GA and **c** fructosamine. Data is presented as Spearman’s correlation coefficient (*r*) and *p*-value. Without CKD, eGFR >60ml/min/1.73m^2^; CKD, eGFR <60ml/min/1.73m^2^

The correlation between FPG and HbA1c, GA, and fructosamine, with the regression line by CKD status, are shown in Fig. 1. In the overall sample (data not shown), FPG was positively associated with HbA1c, GA and fructosamine (*r* = 0.59, *r* = 0.44 and *r* = 0.52, respectively; *p* < 0.0001 for all); with the FPG-HbA1c association being significantly stronger than the FPG-GA (*p* = 0.0062) or FPG-fructosamine association (*p* < 0.0001). When the correlations were analyzed by CKD status, in both groups, FPG was positively associated with HbA1c (*r* = 0.57 and *r* = 0.64, without CKD and with CKD, respectively; *p* < 0.0001 for both), GA (*r* = 0.44 and *r* = 0.51, respectively; both *p* < 0.0001) and fructosamine (*r* = 0.52 and *r* = 0.55, respectively; both *p* < 0.0001 for both), and this association was similar for those with and without CKD (*p* = 0.642; *p* = 0.149 and *p* = 0.312, for HbA1c, GA and fructosamine respectively). Similar results were found in the sub-group of participants with diagnosed diabetes (Appendix Fig. 3). As such, FPG was positively associated with HbA1c, GA and fructosamine in those with and without CKD (*p* < 0.05 for all), with this correlation being similar for people with and without CKD (*p* = 0.158; *p* = 0.274 and *p* = 0.110, for HbA1c, GA and fructosamine respectively).

**Fig. 2:**
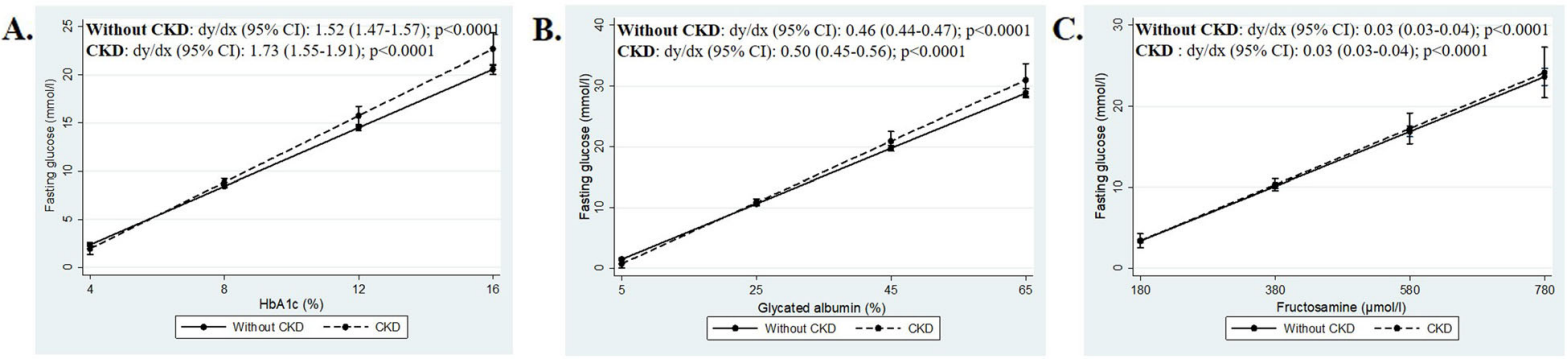
Adjusted association between fasting glucose and markers of glycaemia, **a** HbA1c, **b** glycated albumin, **c** fructosamine, dichotomized by CKD status. Data is presented as (1) linear predictive margins for those with CKD (dashed line) and those without CKD (solid line) with 95% CI and (2) the average marginal effect (dy/dx), 95% CI and *p*-value indicating association between FPG levels and markers of glycaemia, for those with and without CKD

The association between FPG levels and the glycaemic indices, adjusting for age, gender, CKD status and the interaction between CKD status and the glycaemic marker are presented in Table 2, with the interaction dichotomized by CKD status, presented in Fig. 2. Further adjustments for BMI and total haemoglobin or serum albumin are presented in Appendix Table 3, Models 1 and 2, respectively). Higher FPG levels were associated with higher HbA1c, GA and fructosamine levels, independent of age, gender, and CKD status (*p* < 0.0001 for all). Further, the association between FPG and HbA1c as well as GA levels, differed by CKD status (interaction; *p* = 0.030 and *p* = 0.095, respectively), in contrast to the association between FPG and fructosamine, which was similar for those with and without CKD (interaction *p* = 0.851) (Table 2). As such, at HbA1c levels ≥8% and GA levels ≥35%, individuals with CKD had higher FPG than those without CKD (*p* < 0.10) (Fig. 2a and b). Similar results for the association between FPG and HbA1c was found in the sub-group of participants with diagnosed diabetes (interaction; *p* = 0.054), but the FPG-GA and FPG-fructosamine associations were similar for the two groups (interaction; *p* > 0.215 for both) (Appendix Tables 4, 5 and 6, Model 1). Further adjustment of the regression analysis for BMI did not alter the association between FPG and HbA1c, GA or fructosamine (Appendix Table 3, Model 1). In addition, HbA1c and GA were associated with FPG, independent of total haemoglobin and serum albumin, respectively, and adjusting for total haemoglobin had no effect on the effect size of the interaction term CKD*HbA1c. However, when including total serum albumin to the GA model, the effect size of the interaction term CKD*GA was no longer significant (Appendix Table 3, Model 2).

**Table 2:** Adjusted association between fasting glucose and markers of glycaemia (HbA1c, glycated albumin and fructosamine)

	HbA1c			Glycated albumin			Fructosamine		
	β	95% CI	p	β	95% CI	p	β	95% CI	p
Marker of glycaemia	1.52	1.47 to1.57	< 0.0001	0.46	0.44 to 0.47	< 0.0001	0.03	0.03 to 0.04	< 0.0001
Age	−0.04	−0.10 to − 0.01	0.105	− 0.03	−0.02 to 0.09	0.267	−0.03	− 0.10 to 0.10	0.539
Gender	0.05	−0.13 to 0.23	0.579	−0.32	−0.50 to − 0.13	0.001	−0.34	− 0.69 to 0.00	0.052
CKD	−1.22	−2.55 to 0.10	0.071	−1.00	−2.02 to 0.01	0.053	−0.09	−2.04 to 1.86	0.927
CKDxGM	0.21	0.02 to 0.39	0.030	0.05	−0.01 to 0.11	0.095	0.00	−0.01 to 0.01	0.815
Adjusted R^2^	0.72			0.73			0.67		

Data represents β-coefficients, 95% confidence interval, *p*-value and adjusted-R^2^

*CKD* chronic kidney disease, *CKDxGM* interaction between CKD and the respective glycaemic marker, *HbA1c* haemoglobin A1c

## Discussion

The aim of this study was to determine whether the agreement between FPG and markers of chronic glycaemia exposure were affected by reduced kidney function in a mixed-ancestry African population who were not receiving dialysis. This study found that FPG correlated most closely with HbA1c, compared to the alternative markers of chronic glycaemia, however the association between FPG and HbA1c as well as with GA differed by CKD status, particularly at the higher concentration of these markers.

A few studies have explored the association between FPG and measures of chronic glycaemia exposure (HbA1c, GA and fructosamine), with a limited number having investigated this association in those with less severe CKD (stages 3 and 4) [24]. In clinical practice, it is accepted that glycaemic control is best assessed by HbA1c in the general diabetic population. However, studies have demonstrated that HbA1c underestimates and inaccurately reflects long-term glycaemic control in patients with severe CKD, including those with pre-dialysis ESRD [25] and dialysis-dependent CKD [26, 27]. This mechanism for falsely lower HbA1c levels in people with severe CKD can be explained by shortened red blood cell survival in this patient group [7–9]. Yet, what studies do not show is that even during the earlier stages of kidney dysfunction, where the individual might not be aware of their condition, HbA1c assays inaccurately reflect glycaemia. Indeed, in the current study, of which 95% of the participants were in stages 3 and 4 CKD, we found that although FPG correlated well with HbA1c, it underestimated glycaemic control at the higher concentration of the marker (diabetic range) in participants with CKD. This finding was further confirmed in a smaller sub-group of individuals with T2D, in which the adjusted association between FPG and HbA1c differed by CKD status (Appendix Table 4).

Due to the strong link between HbA1c and haemoglobin metabolism, alternative markers of glycaemic control have been proposed for diabetic patients with CKD [25, 28]. These include GA and fructosamine, which have both been shown to accurately reflect glycaemic control [13–15, 25]. Most of these studies were however conducted in patients with CKD receiving either haemodialysis or peritoneal dialysis [13, 27, 29]. It is therefore still not fully known whether these alternative markers are similarly valid to assess glycaemic control in individuals presenting in the earlier stages of kidney dysfunction, prior to receiving dialysis. GA levels are readily influenced by factors associated with albumin turnover [16], and might therefore not appropriately predict glycaemic control in patients with earlier stages of CKD and not on dialysis. Indeed, it has been shown that individuals with CKD, typically with overt albuminuria, have GA values that are lower relative to FPG levels (as found in the current study), typically because of increased albumin metabolism [16]. On the contrary, in patients on dialysis, albuminuria is significantly lower compared to pre-dialysis, potentially mitigating this effect of albumin metabolism [30], thus more accurately reflecting glycaemia in these patients [13, 27, 29]. In addition, a negative association exists between GA and BMI [31, 32], which also potentially affects the usefulness of GA as a marker of glycaemia, particularly with the high global prevalence of obesity [33]. Previous studies have reported lower serum GA levels in both non-diabetic obese and obese T2D patients [31, 32]. In these studies, it was found that GA levels in non-diabetic obese individuals were influenced by factors other than plasma glucose, such as inflammation associated with increased BMI [31]. However, in obese T2D patients GA levels were greatly influenced by insulin levels [34]. Even thought, half the individuals in the current study had a BMI > 30 kg/m^2^, further adjustment of the regression analysis for BMI, did not affect the association between FPG and GA in this sample (Appendix Table 3, Model 1). However, the extent to which BMI affects GA in those with CKD requires further investigation. Fructosamine, has also been proposed as an alternative marker in individuals with CKD, as like GA, it is not affected by haemoglobin-related factors or erythrocyte turnover [28]. However, contradictory results have been reported with respect to the association between FPG and fructosamine in individuals with CKD [35, 36]. Most reported correlation coefficients between FPG and fructosamine, though significant, have been very low and have therefore not allowed fructosamine to be implemented as a reliable marker in glycaemic control. The present study also showed a weaker correlation between FPG and fructosamine, compared to those found for HbA1c and GA. Yet, the relationship between FPG and fructosamine was unaffected by CKD status, portraying it as a potential marker of long-term glycaemic control. With that said, whether fructosamine complements or outperforms HbA1c in individuals with CKD requires further investigation.

Our study has a few limitations, such as the high female to male participation, however this is a common trend in South African population studies, and we do correct for gender in all our analysis. According to NKF-KDOQI guidelines, CKD is defined as an eGFR < 60 ml/min/1.73 m^2^ for ≥3 months and/or increased urinary albumin excretion (≥30 mg/24 h) [21]. For the current study and various other population-based prevalence and association studies in the field of CKD epidemiology, CKD was based on a single time-point creatinine assessment and not on repeated measurements. Further, our study did not include estimates of albuminuria, which is important in the interpretation of eGFR greater that 60 ml/min/1.73m^2^. There were also very few participants in the very advanced stages of CKD (stage ≥4). We also used a single FPG measurement, which is useful for glucose tolerance screening, however for glucose control assessment, serial measurements of blood glucose would have been more appropriate. Other limitations include, small sample size for fructosamine (*n* = 636; 6.8% with CKD) and not measuring potential confounding factors, such as protein and caloric intake. Even though our results should be interpreted cautiously in light of the data limitations, we are not aware of other studies that have assessed the agreement between FPG and HbA1c, GA and fructosamine in individuals with and without CKD, over the complete glycaemic spectrum, in a population-based setting in Africa, specifically individuals of mixed-ancestry. Furthermore, our study consisted of a large sample size and we studied a community with a high burden of obesity and T2D, reflective of the current burden in Africa [37].

## Conclusions

Though HbA1c and GA perform acceptably under conditions of normoglycaemia, our findings suggest that these markers significantly underestimate true glycaemic levels in people with CKD, not on dialysis. Our results suggest that fructosamine may potentially be a more reliable marker of glycaemic levels in those with CKD with elevated FPG. Yet, a limitation to the use of fructosamine as glycaemic marker is that there is no established clinical cut-point for fructosamine and this assay is not standardised across instruments. Therefore, further large-scale studies are needed to demonstrate whether fructosamine has prognostic power to predict adverse clinical outcomes in those with CKD, above that of HbA1c, as there are presently no clinical trial data demonstrating its effectiveness as a glycaemic target in those with moderate CKD.

## Figures and Tables

**Fig. 3:**
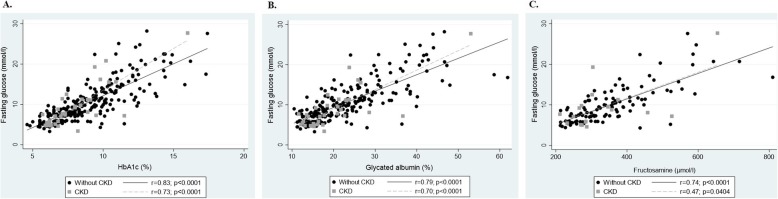
Correlation between fasting glucose, (A) HbA1c, (B) GA and (C) fructosamine in those with confirmed type 2 diabetes. Data is presented as Spearman’s correlation coefficient (*r*) and *p*-value. Without CKD, eGFR >60ml/min/1.73m^2^; CKD, eGFR <60ml/min/1.73m^2^

**Fig. 4:**
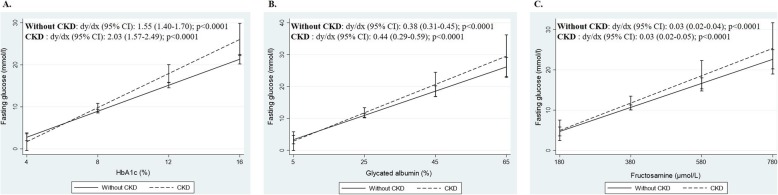
Adjusted association between fasting glucose and markers of glycaemia, (A) HbA1c, (B) glycated albumin, (C) fructosamine, dichotomized by CKD status, in those with confirmed type 2 diabetes. Data is presented as (1) linear predictive margins for those with CKD (dashed line) and those without CKD (solid line) with 95% CI and (2) the average marginal effect (dy/dx), 95% CI and *p*-value indicating association between FPG levels and markers of glycaemia, for those with and without CKD.

## Data Availability

The datasets used and/or analyzed during the current study are available from the corresponding author on reasonable request.

## Appendix

**Table 3:** Adjusted association between fasting blood glucose and markers of glycaemia (HbA1c, glycated albumin and fructosamine)

	MODEL 1			MODEL 2		
	β	95% CI	p	β	95% CI	p
HbA1c	1.51	1.46 to 1.57	< 0.000	1.51	1.46 to 1.56	< 0.0001
Age	−0.00	−0.01 to 0.00	0.093	0.00	−0.01 to 0.00	0.126
Gender	0.07	−0.12 to 0.25	0.471	0.01	−0.18 to 0.20	0.933
CKD	−1.24	−2.56 to 0.08	0.066	−1.25	−2.57 to 0.07	0.064
CKDxHbA1c	0.21	0.21 to 0.40	0.027	0.22	0.03 to 0.40	0.023
BMI	−0.00	− 0.01 to 0.01	0.526	–	–	–
Haemoglobin	–	–	–	0.03	−0.02 to 0.08	0.230
*Adjusted R* ^*2*^	*0.72*			*0.72*		
Glycated albumin	0.45	0.44 to 0.47	< 0.0001	0.46	0.43 to 0.48	< 0.0001
Age	−0.01	−0.06 to 0.04	0.689	0.00	−0.01 to 0.01	0.549
Gender	−0.06	−0.24 to 0.13	0.540	−0.19	− 0.49 to 0.11	0.208
CKD	−1.19	−2.17 to −0.21	0.018	−0.26	− 1.54 to 1.01	0.685
CKDxGA	0.06	0.00 to 0.12	0.033	0.01	−0.06 to 0.08	0.719
BMI	0.04	0.03 to 0.05	< 0.0001	–	–	–
Albumin	–	–	–	−0.04	−0.08 to − 0.00	0.037
*Adjusted R* ^*2*^	*0.74*			*0.71*		
Fructosamine	0.03	0.03 to 0.04	< 0.0001			
Age	−0.07	−0.16 to 0.03	0.167			
Gender	0.01	−0.35 to 0.36	0.970			
CKD	−0.64	−2.53 to 1.25	0.506			
CKDxFructosamine	0.00	−0.00 to 0.01	0.413			
BMI	0.05	0.03 to 0.07	< 0.0001			
*Adjusted R* ^*2*^	*0.69*					

Data represents β-coefficients, 95% confidence interval, *p*-value and adjusted-R^2^. HbA1c, glycated haemoglobin; BMI, body mass index; CKD, chronic kidney disease; CKDxHbA1c; interaction between CKD and HbA1c; CKDxGA; interaction between CKD and glycated albumin; CKDxFructosamine; interaction between CKD and fructosamine; GA, glycated albumin. Model 1: glycaemic marker + age + gender + CKD status + interaction term + BMI; Model 2: glycaemic marker + age + gender + CKD status + interaction term + haemoglobin (HbA1c model) or serum albumin (GA model)

**Table 4:** Adjusted association between fasting blood glucose and HbA1c in those with confirmed type 2 diabetes

	MODEL 1			MODEL 2			MODEL 3		
	β	95% CI	p	β	95% CI	p	β	95% CI	p
HbA1c	1.55	1.40 to 1.70	< 0.0001	1.56	1.41 to 1.71	< 0.0001	1.55	1.40 to 1.70	< 0.0001
Age	− 0.04	−0.07 to − 0.01	0.018	− 0.04	−0.07 to − 0.01	0.014	−0.04	− 0.07 to − 0.01	0.018
Gender	− 0.06	−0.96 to 0.84	0.898	−0.02	− 0.90 to 0.93	0.971	− 0.11	− 1.07 to 0.96	0.830
CKD	−3.00	−7.02 to 1.01	0.142	−2.91	−6.93 to 1.10	0.154	−2.98	−7.00 to 1.04	0.145
CKDxHbA1c	0.47	−0.01 to 0.96	0.054	0.47	−0.01 to 0.95	0.056	0.48	−0.00 to 0.96	0.050
BMI	–	–	–	0.01	−0.04 to 0.06	0.595	–	–	–
Haemoglobin	–	–	–	–	–	–	0.04	−0.19 to 0.28	0.718
Adjusted R^2^	0.66			0.66			0.66		

Data represents β-coefficients, 95% confidence interval, *p*-value and adjusted-R^2^. HbA1c, glycated haemoglobin; BMI, body mass index; CKD, chronic kidney disease; CKDxHbA1c, interaction between CKD and HbA1c. Model 1: HbA1c + age + gender + CKD + CKDxHbA1c; Model 2: Model 1 + BMI. Model 3: Model 1 + haemoglobin

**Table 5:** Adjusted association between fasting blood glucose and glycated albumin in those with confirmed type 2 diabetes

	MODEL 1			MODEL 2			MODEL 3		
	β	95% CI	p	β	95% CI	p	β	95% CI	p
Glycated albumin	0.40	0.35 to 0.44	< 0.0001	0.41	0.36 to 0.45	< 0.0001	0.38	0.31 to 0.45	< 0.0001
Age	−0.05	− 0.08 to − 0.02	0.005	− 0.05	−0.08 to − 0.01	0.006	−0.07	− 0.12 to − 0.02	0.009
Gender	− 0.51	−1.47 to 0.44	0.291	− 0.23	− 1.18 to 0.72	0.635	0.04	− 1.33 to 1.42	0.950
CKD	−1.57	−4.52 to 1.38	0.296	−1.66	−4.54 to 1.23	0.260	−1.79	−4.78 to 3.19	0.695
CKDxGA	0.09	−0.05 to 0.22	0.215	0.09	−0.04 to 0.23	0.169	0.06	−0.10 to 0.23	0.448
BMI	–	–	–	0.07	0.02 to 0.13	0.005	–	–	–
Serum albumin	–	–	–	–	–	–	−0.21	−0.39 to − 0.02	0.027
Adjusted R^2^	0.62			0.64			0.59		

Data represents β-coefficients, 95% confidence interval, *p*-value and adjusted-R^2^. BMI, body mass index; CKD, chronic kidney disease; GA, glycated albumin; CKDxGA, interaction between CKD and GA. Model 1: GA + age + gender + CKD + CKDxGA; Model 2: Model 1 + BMI. Model 3: Model 1 + serum albumin

**Table 6:** Adjusted associations between fasting blood glucose and fructosamine in those with confirmed type 2 diabetes

	MODEL 1			MODEL 2		
	β	95% CI	p	β	95% CI	p
Fructosamine	0.03	0.02 to 0.04	< 0.0001	0.03	0.02 to 0.04	< 0.0001
Age	− 0.08	−0.14 to − 0.03	0.003	−0.07	− 0.13 to − 0.02	0.008
Gender	− 0.08	−1.51 to 1.36	0.916	0.23	−1.19 to 1.65	0.750
CKD	−0.47	−5.61 to 4.67	0.857	−1.39	−6.47 to 3.69	0.589
CKDxFruc	0.00	−0.01 to 0.02	0.586	0.01	−0.01 to 0.02	0.369
BMI	–	–	–	0.08	0.01 to 0.16	0.035
Adjusted R^2^	0.56			0.57		

Data represents β-coefficients, 95% confidence interval, *p*-value and adjusted-R^2^. BMI, body mass index; CKD, chronic kidney disease; CKDxfruc, interaction between CKD and fructosamine. Model 1: fructosamine + age + gender + CKD + CKDxFruc; Model 2: Model 1 + BMI

## Abbreviations

BMI: Body mass index
CKD: Chronic kidney disease
CPUT: Cape Peninsula University of Technology
eGFR: Estimated glomerular filtration rate
ESRD: End-stage renal disease
FPG: Fasting plasma glucose
GA: Glycated albumin
GFR: Glomerular filtration rate
HbA1c: Haemoglobin A1c
IFG: Impaired fasting glucose
IGT: Impaired glucose tolerance
MDRD: Modification of Diet in Renal Disease
NHLS: National Health Laboratory Services
NKF-KDOQI: National Kidney Foundation Disease Outcomes Quality Initiative
OGTT: Oral glucose tolerance test
RBC: Red blood cells
T2D: Type 2 diabetes
VHM: Vascular Metabolic Health
WC: Waist circumference
